# The Effect of the Heating Rate during Carbonization on the Porosity, Strength, and Electrical Resistivity of Graphite Blocks Using Phenolic Resin as a Binder

**DOI:** 10.3390/ma15093259

**Published:** 2022-05-01

**Authors:** Sang-Hye Lee, Jae-Hyun Kim, Woo-Seok Kim, Jae-Seung Roh

**Affiliations:** 1 School of Materials Science and Engineering, Kumoh National Institute of Technology, Gumi 39177, Korea; shlee3106@kumoh.ac.kr; 2 Carbolab Co., Ltd., Gumi 39425, Korea; jhkim@carbolab.co.kr (J.-H.K.); wskim@carbolab.co.kr (W.-S.K.)

**Keywords:** graphite block, phenolic resin, carbonization, porosity, heating rate, microstructure, thermal decomposition, mechanical property, electrical resistivity

## Abstract

In the present study, graphite blocks were fabricated using synthetic graphite scrap and phenolic resin, and the effect of the heating rate during carbonization on their mechanical and electrical characteristics was examined. While varying the heating rate from 1, 3, 5, and 7 to 9 °C/min, the microstructure, density, porosity, flexural strength, compressive strength, and electrical resistivity of the fabricated graphite blocks were measured. As the heating rate increased, the pores in the graphite blocks increased in size, and the shape of the gas release paths became more irregular. Overall, it was found that increases in the heating rate led to the degradation of the graphite blocks’ mechanical and electrical properties.

## 1. Introduction

Recently, carbon materials have been widely used in a range of industrial fields, including the steel, aerospace, and other manufacturing industries [1]. Carbon materials come in different types and shapes, depending on their use, while providing unique properties that cannot be attained with other materials. The demand for these materials will thus continue to expand in the future [2].

Graphite blocks are widely used in various applications, such as steelmaking electrodes, various components of silicon ingot manufacturing equipment, refractories, bearings, special machine parts, and mechanical seals, due to their excellent lubricant properties, heat resistance, corrosion resistance, electrical conductivity, and thermal conductivity [3,4,5].

Graphite blocks are first mixed with fillers and binders and then formed into green bodies through various processes, including uniaxial compression molding, extrusion, and cold isostatic pressing (CIP) [6]. These green bodies are then subjected to carbonization and graphitization to obtain synthetic graphite blocks. Carbonization is the process in which green bodies are heated in an inert or reducing atmosphere. This process is mainly characterized by the dehydrogenation and copolymerization of binders [7]. Notably, in the process, organic materials are decomposed into carbon, and the remaining volatile substances are released into the atmosphere, leaving pores inside the graphite blocks, thereby degrading their material properties [8,9,10].

In general, it is well known that the mechanical properties of these products are the most significantly affected by the microstructure formed during the carbonization process [11,12]. In this regard, the heating conditions during carbonization are considered to be among the most important parameters to determine the properties of graphite blocks because inappropriate heating methods may lead to the formation of pores and cracks. During the heating process, in which binders are converted into carbon-based materials, a large amount of pyrolysis gas is generated, leading to the formation of a porous structure. The formation of this porous structure is mainly governed by both gas generation and diffusion rates, which vary depending on the applied heating rate [13]. Graphite blocks generally fracture in a brittle manner due to the propagation of internal cracks and pores driven by external stress [14]. The strength of isotropic graphite blocks, in particular, is known to be strongly dependent on the size and shape of their internal pores [15].

A significant amount of research has been performed on the effect of carbonization temperatures and heating rates on the thermal properties and mechanical strength of pitch or phenolic resin, a widely used binder for graphite blocks [13,16,17]. However, little research has been focused on the effect of heating rates during the fabrication of graphite blocks on their pore properties and on their electrical and mechanical characteristics. When carbonizing a green body, the composition of the binder material changes more than that of the filler material. During carbonization, the heating rate affects the pyrolysis of the binder and the formation of pores, and it is expected to be related to the change in the physical properties. Therefore, the mechanical and electrical test was conducted with the heating rate as a variable during the carbonization in this study. 

In the present study, the effect of the heating rate during carbonization in the fabrication of synthetic graphite blocks on the formation of internal pores was examined, along with the subsequent effect of the pores’ morphology on the mechanical and electrical properties of the obtained graphite blocks. The research was focused on the changes in the properties of the graphite blocks resulting from the formation of the pores, especially those generated during the volatilization of the binders in the carbonization process. 

## 2. Experimental Procedures

### 2.1. Raw Materials and TG-DTG

Synthetic graphite scrap obtained after processing EDM-3, a product manufactured by POCO Graphite, was used as a filler, and a phenolic resin manufactured by Gangnam Hwaseong (Seoul, Korea) (CB-8081) was used as a binder.

Changes in the weight of this graphite scrap and phenolic resin were measured using TG-DTG analysis (Auto-TGA Q500, TA Instruments, New Castle, DE, USA) while varying the heating rate. The graphite scrap sample was analyzed at a heating rate of 9 °C/min because the synthetic graphite scrap was obtained from a fully graphitized product and, thus, was not expected to undergo weight changes in the process. Phenolic resin, a binder, was heated to 900 °C while varying the heating rate to determine the temperature range in which the maximum weight change would occur. 

### 2.2. Preparation and Carbonization

The filler and the binder were mixed at a weight ratio of 8:2 using a ball mill [18]. The obtained powder mixture was processed into green bodies with dimensions of 10 × 10 × 50 mm using a uniaxial compressor. Subsequently, the obtained green bodies were subjected to carbonization in an inert atmosphere using a tube furnace filled with nitrogen gas. At different heating rates of 1, 3, 5, 7, and 9 °C/min, each sample was heated to 1000 °C and then kept at this temperature for one hour. The obtained carbonized bodies were found to be mostly composed of graphitized synthetic graphite scrap (with a weight ratio of about 90%) and, thus, were named *synthetic graphite* in the present study [19].

### 2.3. Bulk Density and Porosity Measurement

The bulk density and porosity of the graphite blocks were measured using Archimedes’ method (ISO 18754:2012). Five specimens were prepared for each condition, and the dry weight, underwater weight, and saturated weight of the specimens were measured to perform the following calculation.

Bulk density (g/cm^3^) = Dry weight/(Saturated weight − Underwater weight) (1)

Porosity (%) = {(Saturated weight − Dry weight)/(Saturated weight − Underwater weight)} ∗ 100(2)

### 2.4. Microstructural Analysis

The effect of the heating rate on the distribution of pores in the graphite blocks was examined through a microstructural analysis. Each graphite block specimen was finely polished to a surface roughness of 0.25 μm and then observed at a magnification of 200 times using an optical microscope (Nikon ECLIPSE, LV150, Tokyo, Japan).

### 2.5. Flexural and Compressive Strength Measurement

The flexural (10 × 10 × 50 mm) and the compressive (10 × 10 × 25 mm) strength of the specimens were measured in accordance with the standards of ASTM D 7972 and ASTM C 695. For flexural strength measurements, five specimens were prepared and tested for each condition using a universal material tester. The loading point on the upper surface was placed at the midspan, and each of the two loading points on the lower surface was placed 20 mm away from the midspan; the distance between the two points was 40 mm. The loading rate was set to 0.5 mm/min.

S_b_ = 3WI/2bt^2^ (3)

S_b_: Flexural strength (N/cm^2^), I: distance between the two points (cm), W: maximum load (N), b: specimen width (cm), and t: specimen thickness (cm).

For compressive strength measurements, five square pillar-shaped specimens were tested for each condition using a universal material tester. The moving speed of the loading plate was set to 1 mm/min.

Sc = W/S(4)

Sc: Compressive strength (N/cm^2^), W: maximum load (N), and S: area of loading (cm^2^).

### 2.6. Electrical Resistivity Measurement

Electrical resistivity measurements were conducted according to the potential drop method in ASTM C 611. Five specimens (10 × 10 × 50 mm) were prepared and tested for each condition. The potential drop between two voltage terminals, the cross-sectional area of each specimen, the current levels (0.5~3.0 A, 0.5 A interval), and the distance between the two voltage terminals (1.6 cm) were measured to perform the following calculation.

⍴ = eS/il(5)

⍴: Electrical resistivity (Ωcm), e: potential drop between the two terminals (V), S: cross-sectional area of the specimen (cm^2^), i: current (A), and l: distance between the two terminals (cm).

## 3. Results and Discussion

### 3.1. TG-DTG

**Figure 1:**
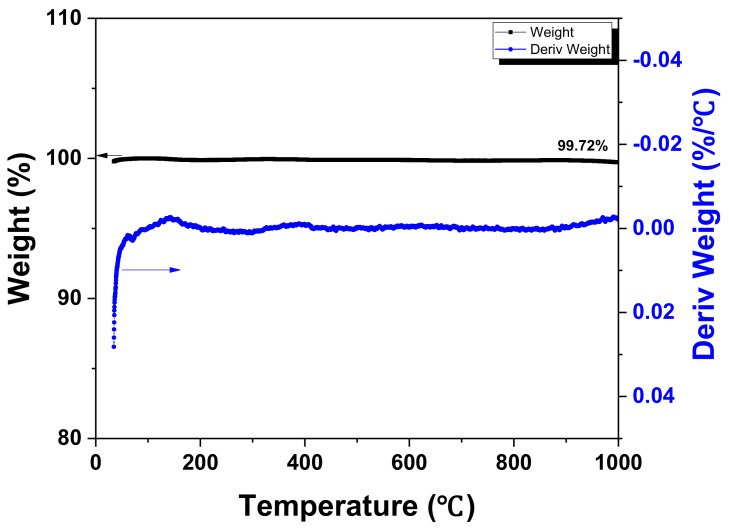
Results of TG−DTG for artificial graphite scrap.

The TG-DTG analysis results of the synthetic graphite scrap used as a filler are presented in Figure 1. After carbonization up to 1000 °C, the carbonization yield was measured as 99.7%, indicating that there was little weight change. The graphite scrap used as a filler in the present study was obtained from a fully graphitized product (possibly around 3000 °C), and, thus, the pyrolysis or release of volatiles no longer occurred below the graphitization temperature. These results confirmed that pores were generated only in the phenolic resin, used as a binder, during the carbonization of the graphite blocks. Accordingly, only the effect of the phenolic resin as a binder was considered in the subsequent analysis of the pores.

**Figure 2:**
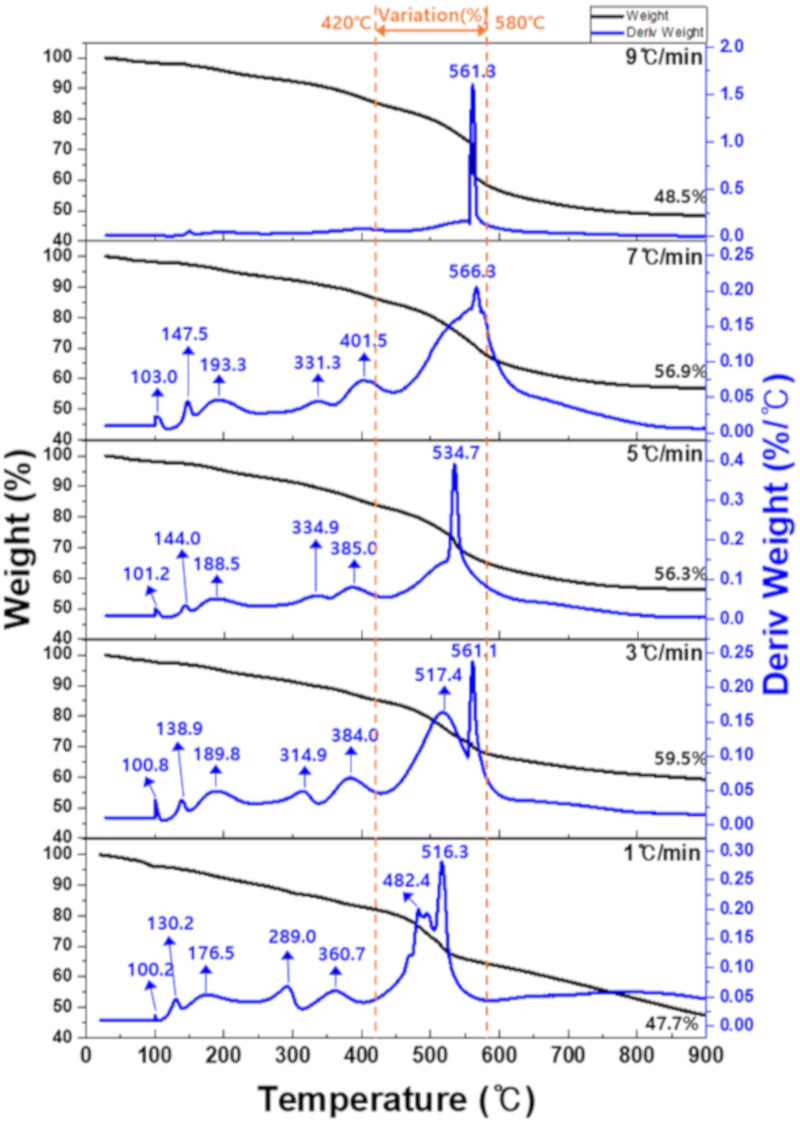
Results of TG-DTG for phenolic resin at heating rates of 1, 3, 5, 7, and 9 °C/min.

The TG-DTG analysis results of the phenolic resin are presented in Figure 2. While the specimen was heated to 900 ℃, continuous slight weight loss was observed. Near 500 °C, in particular, rapid weight change occurred. As shown in the DTG curves, weight loss peaks were observed at 100.2, 130.2, 176.5, 289.0, 360.7, 482.4, and 516.3 °C. In the temperature range up to 400 °C, the weight-loss peaks were found to shift to higher temperatures as the heating rate increased. 

When the heating rate was set to 1 °C/min, the maximum weight-loss peaks were observed at 482.4 and 516.3 °C. When the heating rate was increased to 3 °C/min, these two peaks shifted to higher temperatures, i.e., 517.4 and 561.1 °C, respectively. At the heating rate of 5 °C/min, the two peaks (517.4 and 561.1 °C) began to merge into one, which was observed at 534.7 °C. It was located between 517.4 and 561.1 °C. Subsequently, the peak shifted to a higher temperature at a heating rate of 7 °C/min, and a broad peak was observed at 566.3 °C. When the heating rate was 9 °C/min, a single, sharp weight-loss peak was observed at 563.1 °C. The narrowing of the weight-loss peak can be considered to have occurred more rapidly in a short period of gas release. From this, it was assumed that if the heating rate was higher, the thermal decomposition would change significantly, but further research is needed to confirm this. Similar observations have been reported in previous studies, in which pitch was used as a binder. Bermejo et al. conducted a TG-DTG analysis of coal tar pitch and petroleum pitch while varying the heating rate. The researchers reported that the TG-DTG curves tended to shift to higher temperatures, and the percentage weight loss decreased as the heating rate increased. They attributed this phenomenon to the volatile substances that were not released during heating but trapped in the sample and, accordingly, proposed that low heating rates should be applied during this type of heat treatment [16].

Chang et al. reported that the pyrolysis of phenolic resin exhibited different mechanisms in each of the following three temperature ranges: a low temperature range (<200 °C), an intermediate temperature range (200–600 °C), and a high temperature range (>600 °C). According to the researchers, water evaporated, and phenolic resin was decomposed in the low temperature range, whereas in the intermediate temperature range, CO_2_, CH_4_, and CO gases were released. Finally, in the high temperature range, H_2_ was released. Notably, the temperature range of 420–580 °C is where the rapid release of CO_2_ occurs [20,21,22,23,24].

The carbonization yield and percentage weight loss were measured in the temperature range from 420 to 580 °C, in which the maximum gas release occurred, as shown in Table 1. When the heating rate was set to 1 °C/min and 3 °C/min, the percentage weight loss in the temperate range of 420–580 °C was about 17.5%. When the heating rate was 5 °C/min and 7 ℃/min, the figure was higher, at about 18.6%, indicating an increased amount of gas released. Notably, at the heating rate of 9 °C/min, the percentage weight loss was measured as 26.7%, which confirmed that a very large amount of gas was rapidly released in the process. 

Such a rapid release of gas may lead to the formation of numerous pores during carbonization in the fabrication of graphite blocks. Overall, the TG-DTG analysis results confirmed that the heating rate during carbonization may affect the number of pores generated, demonstrating that the porosity can be controlled by adjusting the heating rate. 

### 3.2. Bulk Density and Porosity Measurement

**Figure 3:**
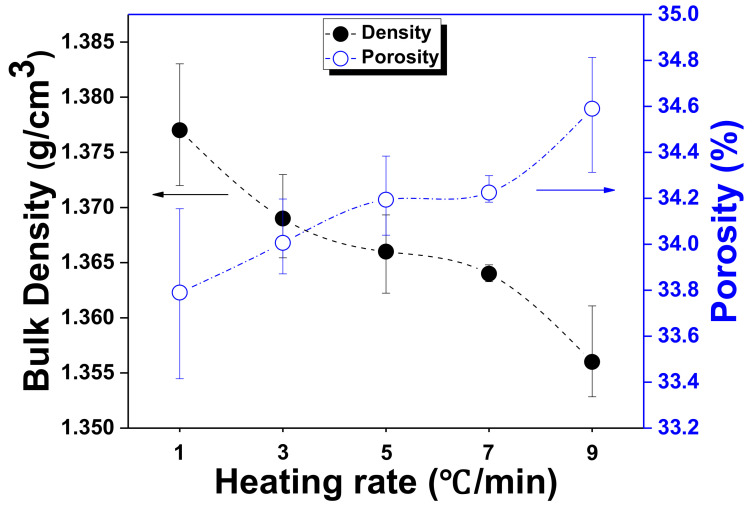
Bulk density and porosity of graphite blocks as a function of heating rate.

The bulk density and the porosity of the graphite blocks were measured as functions of the heating rate, as shown in Figure 3. When the heating rate during carbonization was 1 °C/min, the bulk density of the graphite blocks was the highest, at 1.38 g/cm^3^. The measured bulk densities tended to decrease as the heating rate increased; when the heating rate was 9 °C/min, the bulk density decreased to 1.36 g/cm^3^. The porosity measurements, however, exhibited opposite patterns to those observed in the bulk density results. When the heating rate during carbonization was 1 °C/min, the porosity of the graphite blocks was the lowest, at 38.80%. The measured porosities were found to increase as the heating rate increased; when the heating rate was 9 °C/min, the porosity was the highest, at 39.75%. This was attributed to the greater number of paths for gas release, i.e., pores, required when the heating rate is higher, because the remaining gases must be rapidly released at high temperatures, as previously discussed in the TG-DTG analysis results. 

### 3.3. Microstructure

**Figure 4:**
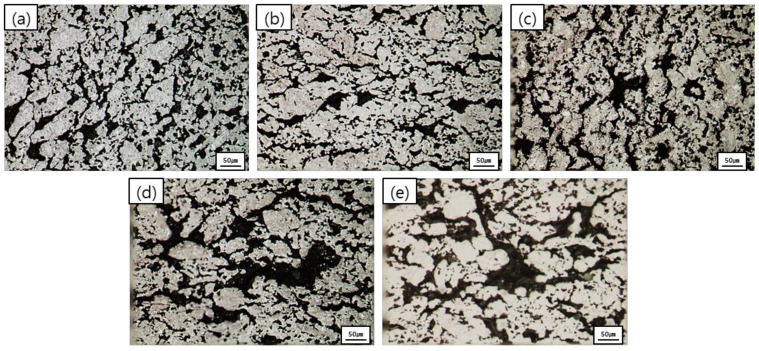
Microstructure of graphite blocks as a function of heating rate (×200) (**a**) 1 °C/min, (**b**) 3 °C/min, (**c**) 5 °C/min, (**d**) 7 °C/min, (**e**) 9 °C/min.

The microstructure images of the graphite blocks with varying heating rates are presented in Figure 4. The results confirmed the formation of pores. When the heating rate was low, fine, spherical pores (about 20~30 µm) with angular shapes were observed in the graphite block specimens (Figure 4a,b). As the heating rate increased, these fine pores started to connect with each other (about 40~70 µm) (Figure 4c) and merge into larger ones (about 60~80 µm in width, and 257 µm in length) (Figure 4d). When the heating rate was 9 °C/min, clusters of large pores (about 75~130 µm in width, and over 258 µm in length) that were interconnected, constituting cracks, were observed (Figure 4e). 

It is worth noting that the porosity increased as the heating rate increased, and that this was because larger pores, which can better serve as paths for gas release, are required, since gases should be more rapidly released at higher heating rates, as previously discussed in the porosity analysis results. The microstructural analysis results confirmed that the higher the heating rate was, the more likely the pores were to merge into larger pores, forming cracks.

### 3.4. Flexural Strength and Compressive Strength

**Figure 5:**
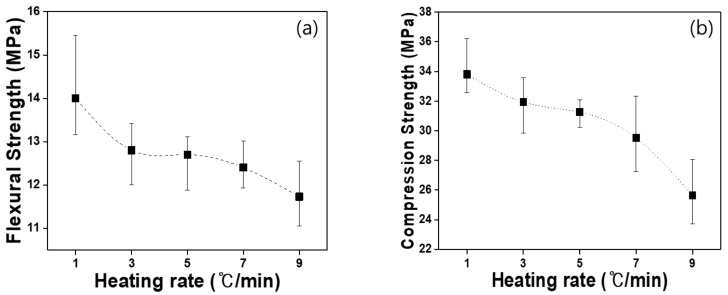
Mechanical strength of graphite blocks as a function of heating rate: (**a**) flexural strength, (**b**) compression strength.

The flexural strengths of the graphite blocks were measured as functions of the heating rate, as shown in Figure 5a. When the heating rate was 1 °C/min, the flexural strength was measured as 13.97 MPa. The measured flexural strength decreased as the heating rate increased; at the heating rate of 9 °C/min, the flexural strength was 11.36 MPa, an 18.7% decrease compared to when the heating rate was 1 °C/min. 

Similarly, the compressive strength was also found to decrease with the increasing heating rate (Figure 5b). When the heating rate was 1 °C/min, the compressive strength was measured as 33.80 MPa. At the heating rate of 9 °C/min, the compressive strength was 25.64 MPa, a 24.1% decrease compared to when the heating rate was 1 °C/min. 

In graphite blocks, the stress is concentrated in the pores. When the stress applied to pores exceeds a certain threshold, the material cannot undergo plastic deformation, where the applied energy can be absorbed and accommodated. This leads to the formation and propagation of cracks [18]. Oshida et al. examined the effect of the size, shape, and number of pores on the flexural strength of isotropic graphite blocks and reported that the mechanical strength of these graphite blocks was highly dependent on the factors described above. In general, the flexural strength of graphite blocks decreases as the number of internal pores increases. According to a previous study, however, when the pores present in graphite blocks are a certain size and distributed in a certain manner, they may hinder the propagation of cracks, thereby helping to absorb the applied stress [25]. Therefore, the porosity is considered to be among the most critical factors determining the mechanical strength of graphite blocks.

As shown in Figure 5, a rapid decrease in the flexural and compressive strengths was observed in the heating-rate range of 5 to 9 °C/min. This result was in line with the TG-DTG, microstructure, and porosity analysis results discussed above. When the heating rate was set to 1 °C/min and 3 °C/min, a rapid release of gas did not occur, and, thus, only fine pores were created in the graphite blocks. When the heating rate was 5 °C/min or more, however, the amount of gas required for release at high temperatures was significantly increased, leading to the merging of the pores, along with the formation of cracks. Once generated, these large pores and cracks considerably reduced the mechanical strength.

As can be seen in Figure 5, the compressive strengths were higher than the flexural strengths, and the strength reduction ratio was higher in the compressive strength measurements. It is worth noting that, in flexural strength tests, cracks tend to propagate along grain boundaries and internal pores before a fracture occurs. Thus, the propagation distance of cracks is relatively short. In compressive strength tests, however, the stress is exerted on the entire top and bottom surface area of the graphite block specimen by the upper and lower punches. Therefore, the pore walls in the graphite block specimen are preferentially broken down. Once the pore walls are broken, the graphite block temporarily becomes denser; however, this is followed by a sudden, rapid increase in stress, finally leading to fracture [26]. This accounts for the measured compressive strengths being higher than the flexural strengths. In addition, as pores increase in size, the effect of increased density is significantly reduced, thereby leading to a large decrease in the mechanical strength.

### 3.5. Electrical Resistivity

**Figure 6:**
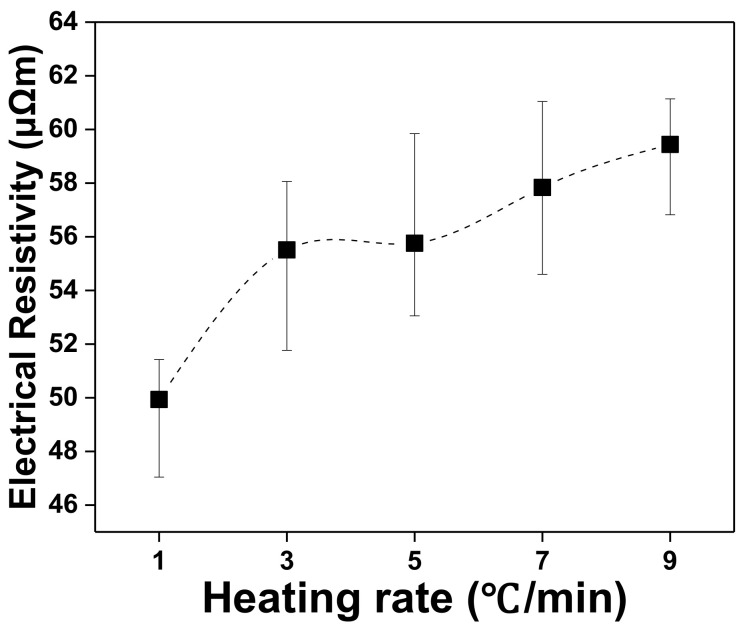
Electrical resistivity of graphite blocks as a function of heating rate.

The electrical resistivities of the graphite blocks were measured and plotted as functions of the heating rate, as shown in Figure 6. When the heating rate was 1 °C/min, the electrical resistivity was measured as 49.96 μΩm. The measured electrical resistivity increased with the increasing heating rate; at the heating rate of 9 °C/min, the electrical resistivity was 59.44 μΩm. It was found that when the heating rate was higher, the internal pores present in the graphite block specimen were more likely to act as barriers, hindering the flow of electrons. A well-known method for reducing the electrical resistivity of materials is to decrease the number of internal pores [27,28]. Overall, it is believed that the electrical properties of materials can be improved by optimizing the heating rate during carbonization to reduce the number of internal pores in them.

## 4. Conclusions

The mechanical and electrical properties of the graphite blocks are presented as a function of the heating rate during carbonization in Table 2. The major findings of the present study are as follows.

First, the phenolic resin, used as a binder, was examined using a TG-DTG analysis to measure the changes in its weight and determine the weight-loss temperature ranges. It was found that at the heating rate of 5 ℃/min, the amount of gas released started to increase. Notably, when the heating rate was 9 ℃/min, a rapid weight change was observed. In addition, as the heating rate increased, the weight-loss temperature ranges (or peaks) tended to shift to higher temperatures, and the corresponding peaks were found to merge into a single peak. This was attributed to the fact that when the heating rate was higher, less time was given for volatiles to be released during heating, and, thus, the increased amount of gas still remaining at high temperatures had to be more rapidly released, leading to the formation of more paths for gas release, i.e., pores.

The microstructural analysis results confirmed that when the heating rate during the carbonization was 5 °C/min or more, large internal pores were formed. Further, these pores tended to merge into larger pores, forming cracks. The size and distribution of the internal pores affected the mechanical and electrical properties of the graphite blocks. More specifically, their flexural strength, compressive strength, and electrical resistivity were lower when the heating rate was higher. Notably, at heating rates of 5 °C/min or more, a significant decrease in these properties was observed. As the heating rate increased (especially at 5 °C/min or more), the internal pores of the graphite blocks tended to increase in size and density due to the rapid release of gas from phenolic resin, and this significantly affected their final properties.

When fabricating a high-density, high-strength graphite block, it is essential to control the pores generated during the carbonization process. It was confirmed that the volatile material and volatilization rate in the phenol are affected by the heating rate during the carbonization process. The heating rate can be used to control the size and shape of the pores formed inside the carbonized body during carbonization. The graphite block carbonization process confirmed that the heating rate leads to the formation of pores inside the carbonized body, and that the pores degrade the mechanical/electrical properties. The porosity increases as the heating rate increases, but the effect of the heating rate has a non-linear relationship with the increasing speed. The difference in the size, shape, and distribution of pores inside the carbonized body is affected by the thermal decomposition rate. At a heating rate higher than a specific level (5 °C/min), the pores expand due to rapid thermal decomposition, and they are interconnected. It is assumed that these interconnected pores caused the decrease in the physical properties in our study. Therefore, when the heating rate is considered as a method of controlling the pyrolysis of the binder, the generation of connected pores may be suppressed. The selection of the heating rate during the carbonization process should be considered among the main variables.

## Figures and Tables

**Table 1:** Carbonization yield and weight loss at 420–580 °C.

Heating Rate (°C/min)	Carbonization Yield (%)		Weight Loss (%) (A−B)
	420 °C (A)	580 °C (B)	
1	82.0	64.4	17.6
3	85.3	67.8	17.5
5	83.9	65.1	18.8
7	86.2	67.8	18.4
9	85.3	58.6	26.7

**Table 2:** Properties of graphite blocks as functions of heating rate.

	Heating Rate				
	1 °C/min	3 °C/min	5 °C/min	7 °C/min	9 °C/min
Bulk density (g/cm^3^)	1.38	1.37	1.37	1.36	1.36
Porosity (%)	33.79	34.01	34.19	34.23	34.59
Flexural Strength (MPa)	13.97	12.78	12.73	12.50	11.36
Compression Strength (MPa)	33.80	31.94	31.26	29.52	25.64
Electrical Resistivity (μΩm)	49.96	55.51	55.76	57.84	59.44
Pore size (µm)	20~30	40~70	40~70	60~80 (width) 257 (length)	75~130 (width) ≥257 (length)

## Data Availability

Data sharing is not applicable.
